# Efficacy and safety of combining radiotherapy with first-line chemotherapy and immunotherapy for local advanced/metastatic urothelial cancer: a propensity score matching analysis

**DOI:** 10.3389/fimmu.2026.1786952

**Published:** 2026-07-08

**Authors:** Jian Qin, Hanjing Zhou, Xia Li, Hubin Yin, Hongbin Deng, Yi Luo, Ying Chen, Yunfei Yin, Rui Zhou, Ni Zhan, Yu Zheng, Tao Zhang, Weiyang He

**Affiliations:** 1 Department of Urology Surgery, The First Affiliated Hospital of Chongqing Medical University, Chongqing, China; 2 Department of Oncology, Laboratory of Immunity, Inflammation and Cancer, The First Affiliated Hospital of Chongqing Medical University, Chongqing, China; 3 Department of Oncology, Chongqing Hospital of Traditional Chinese Medicine, Chongqing, China

**Keywords:** chemotherapy, ICIS, immunotherapy, radiotherapy, UC

## Abstract

**Aim:**

This study evaluates the effectiveness and safety of combining radiotherapy with first-line chemotherapy and immune checkpoint inhibitors in patients with locally advanced or metastatic urothelial carcinoma (la/mUC).

**Methods:**

This retrospective study included patients with la/mUC who received first-line systemic treatment between January 2017 and December 2024. Two treatment strategies were compared: immunotherapy combined with chemotherapy followed by radiotherapy (ICRT group, n = 31) and immunotherapy combined with chemotherapy alone (ICT group, n = 73). Propensity score matching was used to reduce confounding and selection bias, yielding 30 matched patient pairs. Clinical endpoints included objective response rate (ORR), duration of response (DOR), progression-free survival (PFS), overall survival (OS), and treatment-related adverse events.

**Results:**

After propensity score matching, the ICRT group showed significantly prolonged PFS (18.70 vs. 7.93 months; *P* = 0.002) and OS (38.9 vs. 19.8 months; *P* = 0.031) compared with the ICT group. The ICRT cohort demonstrated higher PFS, OS, and ORR across all evaluated time points, with statistically significant differences at 6-month PFS, 12-month PFS, and 12-month OS (*P* < 0.05). Although the median DOR was numerically longer in the ICRT group (29.23 vs. 13.53 months), the difference did not reach statistical significance (*P* = 0.385). Radiation-related enteritis and cystitis were observed in the ICRT group, while no treatment-related deaths occurred. Multivariate Cox regression analysis identified lung metastases, elevated C-reactive protein levels, radiotherapy, and treatment response exceeding stable disease as independent predictors of PFS. Liver metastases, radiotherapy, and better treatment response were independently associated with OS.

**Conclusions:**

The addition of radiotherapy to first-line immunotherapy and chemotherapy demonstrated favorable clinical activity and an acceptable safety profile in patients with la/mUC.

## 1 Introduction

Urothelial carcinoma is the most common histological subtype of bladder cancer and upper tract carcinoma (1). With the steady increase in global life expectancy, the incidence of urothelial carcinoma has continued to increase, and a significant proportion of patients are diagnosed at an advanced stage. For several decades, platinum-based chemotherapy was the standard treatment for locally advanced or metastatic urothelial carcinoma (la/mUC), but the five-year overall survival (OS) rate remained limited to approximately 5–6% (2). In recent years, immune checkpoint inhibitors (ICIs) have progressed from subsequent-line therapies to established first-line treatment options, either as monotherapy or in combination regimens for advanced urothelial carcinoma. These agents have contributed to improved clinical outcomes and offered new therapeutic options for patients with la/mUC.

The KEYNOTE-045 trial (3) demonstrated that second-line PD-1 inhibitor monotherapy was more effective than standard chemotherapy in patients with mUC. Similarly, the KEYNOTE-052 and IMvigor210 studies (4, 5) reported an extension of median OS (mOS) of approximately 1 year with first-line ICI monotherapy in metastatic disease. However, several phase III trials, including KEYNOTE-361, IMvigor130, and DANUBE (6–8), failed to meet their primary endpoints for first-line ICIs, either in combination with chemotherapy or as monotherapy. These findings gradually revealed the limitations of ICIs in the first-line treatment for mUC. In 2020, the JAVELIN Bladder 100 study (9) introduced avelumab as maintenance therapy following first-line platinum-based chemotherapy, achieving a mOS of 23.8 months and establishing a role for maintenance immunotherapy in prolonging survival. Despite this benefit, progression-free survival (PFS) remained modest, with a median PFS (mPFS) of 5.7 months in PD-L1–positive patients and 3.7 months in other subgroups. The CheckMate 901 trial (10) confirmed the efficacy of first-line chemo-immunotherapy, showing a significant improvement in mOS with nivolumab plus chemotherapy compared with chemotherapy alone (21.7 vs. 18.9 months, *P* = 0.02), although the mPFS benefit was limited to 0.3 months compared to the control group. Across first-line studies in la/mUC, ICIs combined with chemotherapy were associated with higher objective response rates (ORRs); however, response durability was frequently insufficient, resulting in a short PFS benefit. More recently, the RC48–016 and EV-302/KEYNOTE-A39 trials (11, 12) reported significant breakthroughs in first-line treatment for mUC. However, limitations related to human epidermal growth factor receptor-2 (HER-2) expression, drug availability, and economic considerations have restricted the broad implementation of these regimens. Effective strategies to extend PFS in la/mUC patients receiving first-line immunotherapy remain an unmet and pressing clinical need.

Primary and acquired resistance pose major challenges to achieving sustained responses with ICIs. Several biological mechanisms contribute to resistance, including expansion of regulatory T cells (Tregs), accumulation of myeloid-derived suppressor cells, loss of tumor antigens, and angiogenesis within the tumor microenvironment (13). Current evidence suggests that combining radiotherapy, chemotherapy, or both with immunotherapy may provide synergistic therapeutic effects by targeting these mechanisms. Radiotherapy induces tumor cell death, promotes intracellular protein degradation, increases antigen availability, and activates the mTOR pathway, which transiently upregulates the surface expression of MHC–peptide complexes on tumor-associated stromal cells (14, 15). It also triggers immunogenic cell death, enhances uptake of tumor-associated antigens, promotes dendritic cell maturation, and stimulates the release of pro-inflammatory cytokines, improving the tumor immune microenvironment and partially overcoming ICI resistance (16, 17). Radiation-induced DNA double-strand breaks in cells activate the cGAS–STING pathway, leading to downstream signaling that increases type I interferon expression and initiates inflammatory cascades, reversing immunosuppression and increasing PD-L1 expression, which may improve responsiveness to ICIs and enhance their efficacy (18). Chemotherapy similarly induces immunogenic cell death, enhances dendritic cell cross-presentation, and promotes CD8^+^ T cell activation selectively, suppresses regulatory T cell expansion, and reduces myeloid-derived suppressor cell populations, synergistically enhancing anti-tumor immune responses (17).

Current clinical investigations have primarily focused on combining radiotherapy or chemotherapy with immunotherapy in patients with inoperable advanced urothelial carcinoma. The IMMUNOPRESERVE, NEXT, ANZUP 1502, BTCRC-GU15-023, and INTACT (S/N 1806) studies (19–23) demonstrated that concurrent or maintenance immunotherapy combined with chemoradiotherapy improved bladder preservation rates and therapeutic efficacy in advanced disease. However, evidence regarding the efficacy and safety of immunotherapy combined with radiotherapy as first-line treatment for la/mUC remains limited. Patients with la/mUC show a higher tendency toward local recurrence after immunotherapy compared with those with early-stage disease. Intrapelvic progression often involves invasion of neuromuscular structures and visceral organs, leading to severe complications such as pain, hemorrhage, edema, fistula formation, and sepsis, which significantly compromise quality of life and may be fatal. Local radiotherapy has the potential to prevent or delay these events. Therefore, this study evaluates the efficacy and safety of local radiotherapy following first-line immunotherapy and chemotherapy, aiming to improve local control, promote organ preservation, relieve primary symptoms, and explore an optimized treatment strategy for la/mUC.

## 2 Objects and methods

### 2.1 Objects

#### 2.1.1 Patients

Patients diagnosed with la/mUC who received first-line platinum-based chemotherapy combined with ICIs at the First Affiliated Hospital of Chongqing Medical University between January 2017 and December 2024 were retrospectively included. According to subsequent treatment strategies, patients were divided into two cohorts: those who received additional radiotherapy after systemic therapy (ICRT group) and those who did not receive radiotherapy (ICT group). This grouping enabled comparison of outcomes between combined modality treatment and immunotherapy–chemotherapy alone.

#### 2.1.2 Inclusion criteria

Patients were eligible for inclusion if they met the following criteria: histological confirmation of urothelial carcinoma according to the AJCC 7th edition staging system; initial systemic treatment consisting of platinum-based chemotherapy combined with ICIs; Eastern Cooperative Oncology Group (ECOG) performance status ≤ 2; estimated glomerular filtration rate (eGFR) ≥ 60 mL/min; recurrent or metastatic lesions confirmed by dynamic imaging, magnetic resonance imaging (MRI), including computed tomography (CT), positron emission tomography/computed tomography (PET/CT), or single-photon emission computed tomography (SPECT); and, for patients with recurrence in the bladder or urinary tract, confirmation by cystoscopic biopsy.

#### 2.1.3 Exclusion criteria

Patients were excluded if they met any of the following conditions: ECOG performance status > 2; presence of other primary malignancies; eGFR < 60 mL/min; or severe uncontrolled comorbid diseases.

### 2.2 Methods

#### 2.2.1 Treatment protocols

All patients with la/mUC received immunotherapy combined with platinum-based chemotherapy as first-line treatment. In the ICRT group, radiotherapy was administered within 2–4 weeks after completion of the last chemotherapy cycle, followed by maintenance immunotherapy every 3 weeks. Patients in the ICT group received 4–6 cycles of combined immunotherapy and chemotherapy, followed by maintenance immunotherapy at 3-week intervals.

Maintenance immunotherapy consisted of PD-1 inhibitors, including pembrolizumab or tislelizumab (200 mg every 3 weeks) and toripalimab (240 mg every 3 weeks), or the PD-L1 inhibitor durvalumab (1500 mg every 3 weeks), for a maximum of 35 cycles. Upon disease progression, subsequent treatment strategies were selected based on individual clinical conditions and included surgical resection, second-line cytotoxic chemotherapy, radiotherapy, antibody–drug conjugates, continued immune checkpoint inhibition, multi-kinase inhibitors, or best supportive care.

#### 2.2.2 Observation indicators

Peripheral blood samples were collected within 3 days before initiation of first-line treatment to determine neutrophil count, lymphocyte count, neutrophil-to-lymphocyte ratio (NLR), C-reactive protein (CRP), and lactate dehydrogenase (LDH) levels. Contrast-enhanced CT of the chest, abdomen, pelvis, and metastatic sites with urinary tract computed tomography urography when indicated, was performed every 2–3 months during follow-up. Therapeutic efficacy was assessed according to the Response Evaluation Criteria in Solid Tumors (RECIST) version 1.1.

#### 2.2.3 Statistical analysis

To reduce potential confounding and selection bias, propensity score matching (PSM) was performed between the two groups. Clinical variables incorporated into the PSM model included age, ECOG performance status, smoking history, recurrence status, metastatic sites (liver, lung, and bone), CRP level, and response to chemotherapy (complete remission [CR] or partial remission [PR]). Matching was conducted using a nearest-neighbor algorithm with a 1:1 ratio.

Comparisons between groups were performed using Student’s *t*-test for continuous variables and the chi-square test for categorical variables. Statistical analyses were carried out using SPSS software (version 26.0; IBM) and R software (version 4.5.0; R Foundation for Statistical Computing). Survival analyses were initially conducted using the Kaplan–Meier method, with differences evaluated by the log-rank test. Variables with *P* < 0.1 in univariate analysis were entered into multivariate Cox proportional hazards regression models to identify independent predictors of OS and PFS. All statistical tests were two-sided, and *P* < 0.05 was considered statistically significant.

## 3 Results

### 3.1 Baseline characteristics

A total of 115 patients were screened for eligibility, of whom 31 were assigned to the ICRT group and 73 to the ICT group. Comparative analysis identified significant differences between the two groups (*P* < 0.05) in smoking history, incidence of bone metastasis, CRP levels, and CR or PR rates following chemotherapy. No statistically significant differences (*P* > 0.05) were observed for age, sex, ECOG performance status, oligometastatic disease, presence of lung or liver metastases, NLR, or LDH levels. Among 104 evaluable patients, PD-L1 testing was performed in 8 cases, with PD-L1 status remaining undetermined in the remaining patients. Human epidermal growth factor receptor 2 (HER2) testing was performed in 16 patients; 6 showed HER2 (++/+++), whereas the remaining 10 showed HER2 (+). After PSM, baseline clinical characteristics were more balanced between the two groups (**Table 1**).

**Table 1:** Baseline characteristics of all patients.

Characteristic	Before matching			After matching		
	Radiation Group	PD-1 Group	*P* value	Radiation Group	PD-1 Group	*P* value
	(n=31)	(n=73)		(n=30)	(n=30)	
Age (x ± s, years)	65.71 ± 9.54	68.22 ± 11.34	0.283	65.70 ± 9.70	68.43 ± 12.11	0.339
Gender (female/male)	10/21	14/59	0.148	10/20	4/26	0.067
ECOG (0-1/2)	22/9	49/24	0.700	21/9	17/13	0.284
Smoke (≤400/>400)	15/16	56/17	0.005	15/15	20/10	0.190
Oligometastatic disease (Multiple/Oligo)	9/15	13/31	0.503	9/14	5/15	0.324
Pulmonary metastasis (No/Yes)	24/7	59/14	0.693	23/7	24/6	0.754
Bone metastasis (No/Yes)	19/12	62/11	0.008	19/11	23/7	0.260
Postoperative recurrence (No/Yes)	8/23	23/50	0.561	8/22	12/18	0.273
Liver metastasis (No/Yes)	25/6	67/6	0.175	24/6	28/2	0.254
Nodel metastasis (No/Yes)	18/13	53/20	0.145	17/13	23/7	0.100
CRP (x ± s, mg/L)	6.81 ± 6.02	11.92 ± 18.23	0.035	6.27 ± 5.31	12.10 ± 23.67	0.197
LDH (x ± s, U/L)	202.55 ± 178.68	221.81 ± 162.35	0.592	204.87 ± 181.26	210.13 ± 122.62	0.896
NLR (x ± s)	7.83 ± 10.14	4.43 ± 5.40	0.086	6.86 ± 8.71	4.90 ± 6.62	0.330
Chemotherapy response to CR/PR (No/Yes)	16/15	57/16	0.007	15/15	20/10	0.190

The study population received PD-1 or PD-L1 inhibitor therapy, including tislelizumab (n = 63), toripalimab (n = 27), pembrolizumab (n = 11), and durvalumab (n = 3), with a median treatment duration of 5.25 months, corresponding to seven cycles. Chemotherapy regimens consisted of taxane-based agents, gemcitabine, and platinum compounds, with a median of 3 first-line chemotherapy cycles per patient. In the radiotherapy cohort, irradiation sites included osseous metastases (n = 6), cerebral lesions (n = 1), pelvic or bladder tumors with or without extended pelvic field irradiation (n = 21), and nodal metastases (n = 3). Consolidative radiotherapy was administered to 13 patients, including six with oligometastatic disease and seven with local recurrence. In most cases, all identifiable lesions were irradiated; however, one patient with oligometastatic disease received irradiation to only a subset of lesions. Among the 18 patients who underwent palliative radiotherapy, the number of oligometastatic and polymetastatic cases was equal. Radiotherapy was primarily directed at symptomatic lesions, and only selected metastatic sites were treated, except in four patients with bone metastases who received more comprehensive irradiation. Radiotherapy target delineation comprised the primary gross tumor volume (GTVp), with or without the pelvic clinical target volume (CTVpelvic) for locally advanced urothelial carcinoma; the metastatic gross tumor volume (GTVm) for distant lesions; and elective nodal irradiation (ENI) for involved lymph nodes. The CTVpelvic included the entire bladder, the prostatic urethra in male patients, and the obturator and iliac lymph node regions, covering both internal and external iliac chains. Fractionation schedules ranged from 1.8 to 5 Gy per fraction, with total prescribed doses of 30–70 Gy delivered to GTV, ENI, or CTV, corresponding to biological effective doses (BED) of 37.5–84 Gy (**Table 2**).

**Table 2:** Radiation dose and fractions of the radiation group.

	Physical dose (Gy)	Biological Effect dose (Gy)	Local Control rate (%)
Conventional radiation for relapse pelvic lesions (6 cases)	50-70Gy/25-35F/2Gy	60-84Gy	33.3%
Hypo-fractionated radiation for relapse pelvic lesions (1 case)	25Gy/5F/5Gy	37.5Gy	100%
Conventional radiation for pelvic/bladder lesions + small pelvic field irradiation (13 cases)	GTV 60-68.16Gy/30-32F/2-2.13Gy CTVpv 45-50.04Gy/25-28F/1.8-2Gy	72-82.67Gy	76.9%
Elective Nodal radiation (3 cases)	60Gy/30F/2Gy	72Gy	100%
Conventional radiation for Bone metastases (2 cases)	50Gy/25F/2Gy	60Gy	50%
Hypo-fractionated radiation for Bone metastases (4 cases)	30Gy/10F/3Gy	39Gy	100%
Hypo-fractionated radiation for Brain metastases (1 case)	45Gy/15F/3Gy	58.5Gy	NA

### 3.2 Effectiveness and safety

At the final follow-up, 87.5% of patients experienced disease progression, and 56.7% reached the endpoint of OS. Among all 104 evaluable patients, the mPFS was 9.3 months, the median duration of response (mDOR) was 22.1 months, and the ORR was 29.8%.

**Figure 1:**
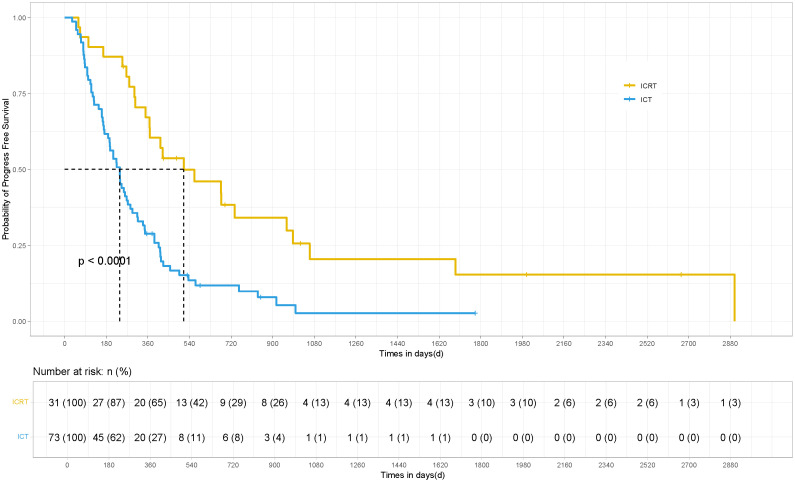
Progression-free survival (PFS) of patients in the ICRT and ICT groups (n = 104). The median PFS (mPFS) was significantly longer in the ICRT group (17.17 months) than in the ICT group (7.93 months), with a hazard ratio (HR) of 0.42 (95% CI, 0.282–0.642; *P* < 0.001).

**Figure 2:**
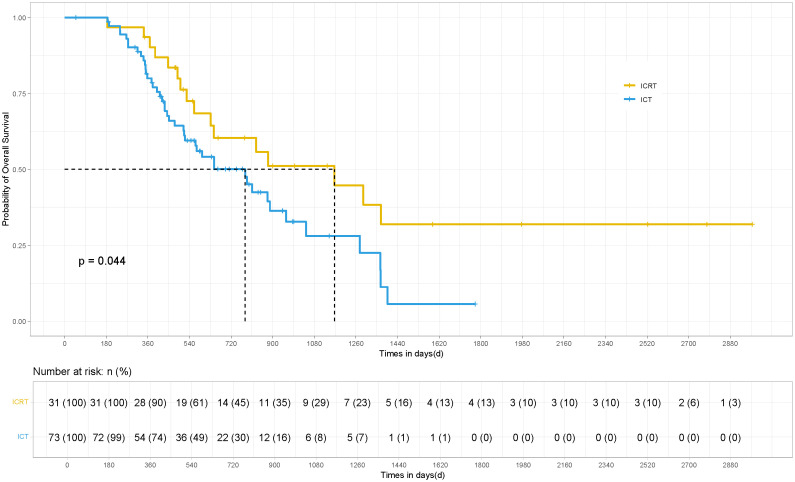
Overall survival (OS) of patients in the ICRT and ICT groups (n = 104). The median OS (mOS) was significantly prolonged in the ICRT group (38.9 months) compared with the ICT group (26.03 months), with an HR of 0.57 (95% CI, 0.342–0.971; *P* = 0.044).

In the unmatched analysis before PSM, the ICRT group showed significantly better clinical outcomes than the ICT group. The mPFS was significantly longer in the ICRT group than in the ICT group (17.17 vs. 7.93 months; hazard ratio 0.42; *P* < 0.001) (**Figure 1**). The mOS was also prolonged in the ICRT group compared with the control group (38.9 vs. 26.03 months; hazard ratio 0.57; *P* = 0.044) (**Figure 2**). The ICRT group maintained higher PFS rates at all evaluated time points, including 3 months (93.5% vs. 83.6%), 6 months (87.1% vs. 61.6%), and 12 months (67.1% vs. 28.8%), with statistically significant differences observed at 6 and 12 months (*P* < 0.05). OS rates similarly favored the radiotherapy cohort at 1 year (93.5% vs. 80.0%), 2 years (60.4% vs. 50.1%), and 3 years (51.1% vs. 28.1%), although statistical significance was reached only at the 3-year time point (*P* < 0.05) (**Table 3**). Response assessment showed comparable CR rates between groups (3.2% vs. 4.1%, *P* = 1.00), whereas the ORR was significantly higher in the ICRT group than in the ICT group (48.4% vs. 21.9%, *P* = 0.007), with 15 responders (one CR and fourteen PRs) in the ICRT group and 16 responders in the ICT group.

**Table 3:** Survival analysis and therapeutic efficacy of the two treatment groups.

Index	Before matching			After matching		
	Radiation Group	PD-1 Group	*P* value	Radiation Group	PD-1 Group	*P* value
	(n=31)	(n=73)		(n=30)	(n=30)	
mPFS (month) (95% CI)	17.17 (6.55-27.79)	7.93 (6.38-9.48)	<0.001	18.70 (5.61-31.79)	7.93 (4.98-10.88)	0.002
PFS rate (%)						
3 month	93.5	83.6	0.221	93.3	86.7	0.671
6 month	87.1	61.6	0.010	90.0	63.3	0.015
12 month	67.1	28.8	<0.001	69.3	30.0	0.003
mOS (month) (95% CI)	38.90 (17.62-60.19)	26.03 (18.46-33.60)	0.044	38.90 (20.62-57.18)	19.80 (6.03-33.57)	0.031
OS rate (%)						
12 month	93.5	80.0	0.137	96.7	71.8	0.012
24 month	60.4	50.1	0.205	62.4	46.4	0.322
36 month	51.1	28.1	0.026	52.8	29.7	0.078
m Dor (month) (95% CI)	29.23 (7.82-50.64)	13.53 (6.16-20.90)	0.138	29.23 (7.82-50.64)	13.53 (0.00-31.09)	0.385
Efficacy (cases)						
CR	1	3	1.000	1	2	1.000
PR	14	13	0.004	14	8	0.127
SD	13	43	0.748	12	14	0.700
ORR (%)	48.4	21.9	0.007	48.4	33.3	0.190
DCR (%)	90.3	80.8	0.384	90.0	80.0	0.472

**Figure 3:**
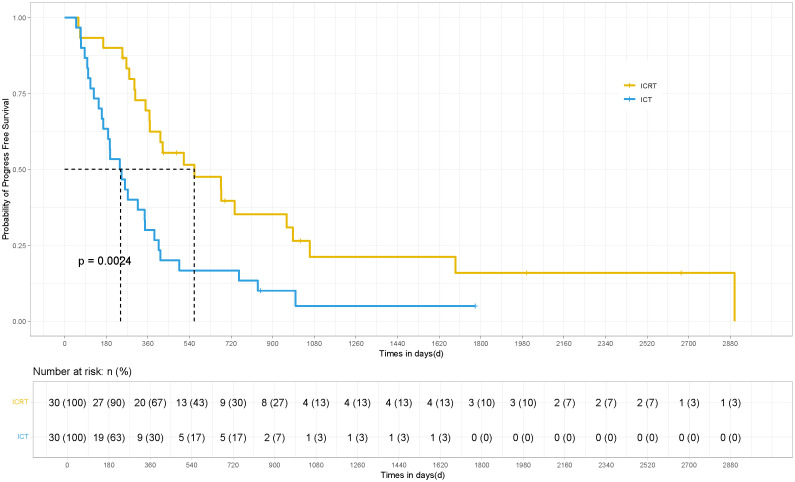
Progression-free survival (PFS) of patients in the ICRT and ICT groups (n = 60). The mPFS was significantly longer in the ICRT group (18.70 months) than in the ICT group (7.93 months), with an HR of 0.44 (95% CI, 0.226–0.714; *P* = 0.002).

**Figure 4:**
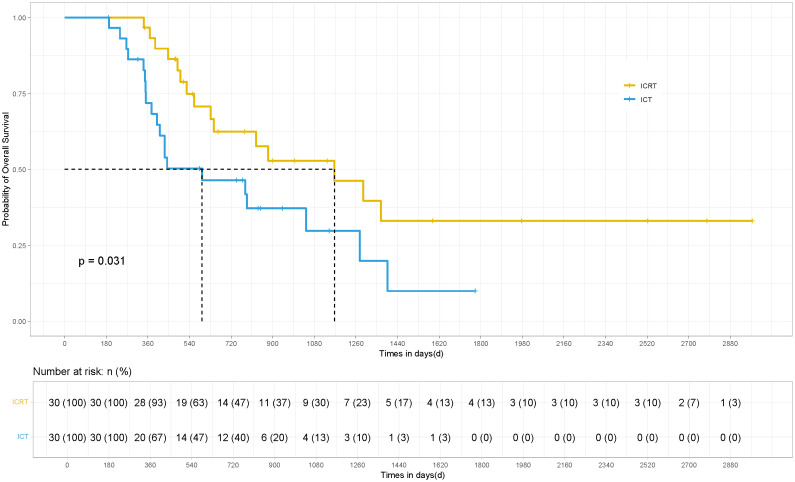
Overall survival (OS) of patients in the ICRT and ICT groups (n = 60). The mOS was significantly longer in the ICRT group (38.9 months) than in the ICT group (19.8 months), with an HR of 0.49 (95% CI, 0.239–0.929; *P* = 0.031).

After PSM, the ICRT cohort continued to show better clinical efficacy compared with the control group. The mPFS remained significantly prolonged in the ICRT group (18.70 vs. 7.93 months; hazard ratio 0.44, 95% confidence interval 0.226–0.714, *P* = 0.002), as did mOS (38.9 vs. 19.8 months; hazard ratio 0.49, 95% confidence interval 0.239–0.929, *P* = 0.031) (**Figures 3**, **4**). PFS rates were higher in the ICRT group at 3 months (93.3% vs. 86.7%), 6 months (90.0% vs. 63.3%), and 12 months (69.3% vs. 30.0%), with statistically significant differences at 6 and 12 months (*P* < 0.05). Although OS rates from 1 to 3 years were numerically higher in the ICRT group, only the 1-year difference reached statistical significance (*P* < 0.05) (**Table 3**). No statistically significant differences were observed between groups in mDOR (29.23 vs. 13.53 months, *P* = 0.385) or ORR (48.4% vs. 33.3%, *P* = 0.190) after matching.

In terms of disease progression patterns, 23 patients in the radiotherapy group experienced progression events, of whom eight showed recurrence within irradiated fields; one patient with brain metastasis failed to complete the planned radiotherapy. The local control rate (LCR) in the ICRT group was 73.4%, with a high first-year local control probability of 86.7%. On the other hand, 28 patients in the ICT group experienced disease progression, nearly half of whom (13 cases, 43.3%) showed local recurrence, with 76.9% occurring within the first year of treatment at primary tumor sites. The first-year LCR was significantly lower in the ICT group than in the ICRT group (23.1% vs. 86.7%). Before treatment, 30 patients reported symptoms such as pain, urinary urgency, and hematuria. Following radiotherapy, symptom relief was achieved in 85.7% of these patients.

**Figure 5:**
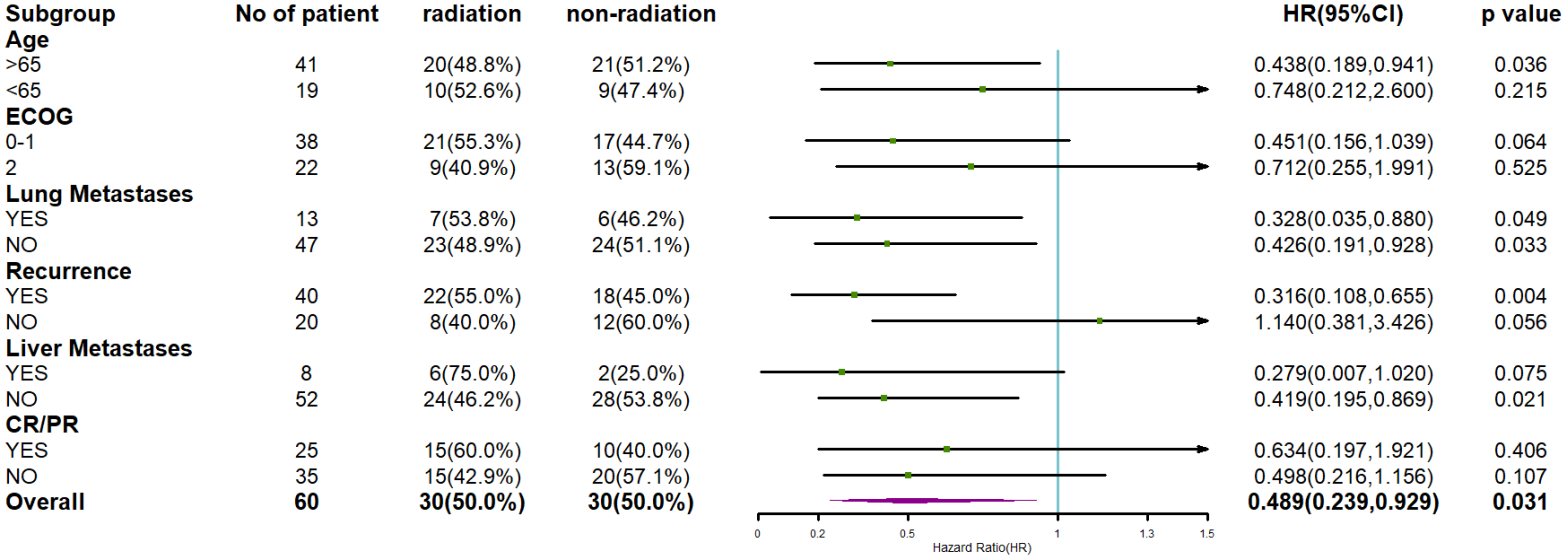
Subgroup analysis of overall survival (OS). Subgroup analysis demonstrated that the combination of radiotherapy and immune checkpoint inhibitors conferred better survival benefits in specific patient populations, including individuals aged 65 years or older, patients without lung or liver metastases, and those with postoperative disease recurrence.

Subgroup analyses identified significant OS benefit from the combination of ICIs and radiotherapy in patients older than 65 years (*P* = 0.036), those without lung (*P* = 0.033) or liver metastases (*P* = 0.021), and patients with postoperative recurrence (*P* = 0.004). A trend toward improved OS with the addition of radiotherapy was also observed in patients with ECOG performance status 0–1 (*P* = 0.064) and in those who did not achieve CR or PR after initial systemic therapy (*P* = 0.107), suggesting potential benefit in these clinically relevant subgroups (**Figure 5**).

Regarding safety, the most frequently reported treatment-emergent adverse events were chemotherapy-related hematologic toxicities, including leukopenia, thrombocytopenia, and anemia. No treatment-related deaths occurred in either group. Other adverse events included elevated hepatic transaminases (3 cases), thyroid dysfunction (1 case), ICI-associated myocarditis (2 cases), immune-related myelitis (1 case), and rash (1 case). No statistically significant differences in treatment-related adverse events were observed between the two groups (**Table 4**). The ICRT group experienced radiotherapy-specific toxicities, including radiation cystitis (4 cases) and radiation enteritis (4 cases), all occurring within 6 months after radiotherapy. Only one case of Grade 3 radiation cystitis was reported, which resolved following comprehensive management with corticosteroids, antimicrobial therapy, nutritional support, and symptomatic treatment.

**Table 4:** Safety assessment of the two treatment groups.

Toxic effects	Total (n=60)	Radiation Group (n=30)	PD-1 Group (n=30)	*p* value
Leukopenia	18	9	9	1.000
Grade 1-2/case	4	2	2	
Grade 3-4/case	14	7	7	1.000
Thrombocytopenia	20	10	10	1.000
Grade 1-2/case	8	3	5	
Grade 3-4/case	12	7	5	0.650
Anemia	11	6	5	0.739
Grade 1-2/case	7	3	4	
Grade 3-4/case	4	3	1	0.545
Hepatocyte dysfunction	3	2	1	1.000
Thyroid dysfunction	1	1	0	1.000
Cardiotoxicity	2	2	0	0.492
Myelitis	1	0	1	1.000
Pneumonia	0	0	0	1.000
Drug-induced rash	1	0	1	1.000
Radio-cystitis	4	4	/	0.112
Grade 1-2/case	3	3	/	0.237
Grade 3-4/case	1	1	/	1.000
Radiation enteritis	4	4	/	0.112

Both groups showed comparable safety profiles. Hematologic toxicities, including chemotherapy-associated leukopenia, thrombocytopenia, and anemia, were the most frequently observed adverse events. No treatment-related deaths occurred in either group. The incidence of treatment-emergent adverse events, including elevated hepatic transaminases, thyroid dysfunction, immune checkpoint inhibitor–associated myocarditis, myelitis, and cutaneous reactions, did not differ significantly between groups. Patients in the ICRT group experienced radiation-related toxicities, such as cystitis and enteritis; all were manageable with appropriate clinical intervention.

### 3.3 Factors affecting PFS and OS

Univariate analysis was first conducted to assess the association between clinical variables and PFS. Variables included patient demographics (age and gender), performance status (ECOG score), smoking history, recurrence status, metastatic sites (lung, bone, lymph node, and liver), metastatic status (polymetastatic or oligometastatic disease), serum biomarkers (CRP, LDH, and NLR), radiotherapy administration, and response to first-line chemotherapy. This screening identified seven variables that showed significant or borderline associations (*P* < 0.1) with PFS: gender (*P* = 0.043), ECOG performance status (*P* = 0.09), lung metastases (*P* = 0.067), CRP level (*P* < 0.001), objective response to first-line treatment (*P* < 0.001), radiotherapy administration (*P* = 0.003), and oligometastatic disease (*P* = 0.024).

These variables were then included in a multivariate Cox proportional hazards regression model using the enter method. Four independent prognostic factors for PFS were identified. The presence of lung metastases was associated with increased risk of progression (regression coefficient 0.757, Wald value 4.138, *P* = 0.041; hazard ratio 2.133, 95% confidence interval 1.028–4.425). An elevated CRP level was also associated with poorer PFS (regression coefficient 0.02, Wald statistic 5.953, *P* = 0.015; hazard ratio 1.021, 95% confidence interval 1.004–1.037). Radiotherapy was associated with a reduced risk of progression (regression coefficient −1.229, Wald value 13.373, *P* < 0.001; hazard ratio 0. 293, 95% confidence interval 0.151–0.565), as was achievement of an objective response to first-line treatment (regression coefficient −1.785, Wald value 22.297, *P* < 0.001; hazard ratio 0.168, 95% confidence interval 0.08–0.352). (**Table 5**).

**Table 5:** Prognostic factors for progression-free survival in patients with locally advanced or metastatic urothelial carcinoma.

Characteristic	Univariate analysis			Multivariate analysis		
	HR	95% CI	*P* value	HR	95% CI	*P* value
Age	1.012	0.986-1.038	0.386			
Sex						
Male	1			1		
Female	2.186	1.025-4.664	0.043	1.580	0.695-3.592	0.275
ECOG						
0-1	1			1		
2	0.611	0.346-1.080	0.090	1.269	0.674-2.389	0.460
Smoke						
>400	1					
≤400	1.273	0.728-2.226	0.397			
Pulmonary metastasis						
Yes	1			1		
No	1.819	0.958-3.454	0.067	2.133	1.028-4.425	0.042
Bone metastasis						
Yes	1					
No	1.173	0.647-2.129	0.599			
Liver metastasis						
Yes	1					
No	1.303	0.610-2.783	0.494			
Nodel metastasis						
Yes	1					
No	1.003	0.557-1.806	0.993			
Postoperative recurrence						
Yes	1					
No	0.821	0.455-1.483	0.513			
CRP	1.029	1.012-1.045	< 0.001	1.021	1.004-1.037	0.015
LDH	1.001	0.999-1.003	0.286			
NLR	1.008	0.971-1.046	0.679			
Chemotherapy response to CR/PR						
Yes	1			1		
No	0.209	0.110-0.395	< 0.001	0.168	0.080-0.352	< 0.001
Oligometastatic disease						
Recurrence only	1			1		
Oligometastatic	1.373	0.685-2.753	0.372	0.988	0.444-2.2	0.977
Polymetastatic	2.456	1.126-5.359	0.024	2.434	0.991-5.979	0.052
Radiotherapy						
Yes	1			1		
No	0.425	0.241-0.750	0.003	0.293	0.151-0.565	< 0.001

HR hazard ratio, CI confidence interval, ECOG Eastern Cooperative Oncology Group performance status, CRP C-reactive protein, LDH lactate dehydrogenase, NLR neutrophil-to-lymphocyte ratio.

For OS, univariate screening identified several variables with potential prognostic relevance (*P* < 0.1), including age (*P* = 0.070), liver metastases (*P* = 0.098), CRP level (*P* = 0.008), treatment response (CR or PR, *P* = 0.001), radiotherapy (*P* = 0.035), and metastatic status (*P* = 0.018). These factors were entered into a multivariate Cox regression model. Three variables remained independently associated with OS. The presence of liver metastases significantly increased the risk of death (regression coefficient 1.287, Wald value 4.095, *P* = 0.043; hazard ratio 3.621, 95% confidence interval 1.041–12.59). Radiotherapy was associated with improved OS (regression coefficient −1.047, Wald value 6.812, *P* = 0.009; hazard ratio 0. 351, 95% confidence interval 0.16–0.77), and achievement of CR or PR similarly showed a protective association (regression coefficient −1.315, Wald value 9.219, *P* = 0.002; hazard ratio 0.268, 95% confidence interval 0.115–0.627). (**Table 6**).

**Table 6:** Prognostic factors for overall survival in patients with locally advanced or metastatic urothelial carcinoma.

Characteristic	Univariate analysis			Multivariate analysis		
	HR	95% CI	*P* value	HR	95% CI	*P* value
Age	1.031	0.997-1.065	0.070	1.040	1.000-1.082	0.052
Sex						
Male	1					
Female	1.826	0.755-4.416	0.181			
ECOG						
0-1	1					
2	0.684	0.348-1.345	0.271			
Smoke						
>400	1					
≤400	1.082	0.553-2.115	0.818			
Pulmonary metastasis						
Yes	1					
No	1.791	0.847-3.789	0.127			
Bone metastasis						
Yes	1					
No	1.247	0.619-2.514	0.537			
Liver metastasis						
Yes	1			1		
No	2.021	0.878-4.650	0.098	3.621	1.041- 12.590	0.043
Nodel metastasis						
Yes	1					
No	1.031	0.511-2.081	0.931			
Postoperative recurrence						
Yes	1					
No	0.711	0.355-1.423	0.335	1.014	0.995-1.034	0.154
CRP	1.023	1.006-1.040	0.009			
LDH	1.001	0.999-1.003	0.409			
NLR	1.024	0.985-1.064	0.237			
Chemotherapy response to CR/PR						
Yes	1			1		
No	0.286	0.135-0.607	0.001	0.268	0.115-0.627	0.002
Oligometastatic disease						
Recurrence only	1			1		
Oligometastatic	1.469	0.603-3.581	0.398	2.238	0.797-6.282	0.126
Polymetastatic	3.138	1.219-8.079	0.018	3.082	0.879-10.803	0.079
Radiotherapy						
Yes	1			1		
No	0.483	0.245-0.949	0.035	0.351	0.160-0.770	0.009

HR hazard ratio, CI confidence interval, ECOG Eastern Cooperative Oncology Group performance status, CRP C-reactive protein, LDH lactate dehydrogenase, NLR neutrophil-to-lymphocyte ratio.

## 4 Discussion

In recent years, ICIs have rapidly advanced as first-line treatment options for mUC, producing promising therapeutic outcomes. However, the ORR of ICI monotherapy in metastatic disease remains less than 30%. Clinical trials have shown that combining chemotherapy with ICIs increases ORR to approximately 40–57.6% (24). Despite this improvement, PFS benefits remain limited. The EV-302/KEYNOTE-A39 and RC48–016 studies confirmed significant clinical improvements in the first-line treatment of mUC. Although these landmark studies represent important advances, their broad application remains limited by requirements for HER2 expression, drug availability, and economic considerations. In routine clinical practice, patients with mUC frequently experience a high risk of local disease progression following systemic therapy, leading to short DOR, particularly in those with poor initial treatment response. Local recurrence is often accompanied by symptoms such as pain, hematuria, and anemia, which significantly reduce quality of life. Therefore, there is an urgent need for practical treatment strategies that extend PFS in the first-line treatment, reduce local recurrence, and improve patient-reported outcomes. Among current investigational approaches, the integration of radiotherapy with immunotherapy has emerged as a particularly promising therapeutic option. In urothelial carcinoma, radiotherapy has an established role in bladder-preservation strategies for advanced disease and as consolidative treatment for recurrent or metastatic disease following systemic therapy.

In muscle-invasive bladder cancer (MIBC), trimodality therapy (TMT) offers a bladder-preserving alternative to radical cystectomy. The BTCRC-GU15–023 study combined transurethral resection of bladder tumor with durvalumab and radiotherapy, reporting 73% and 83.8% one-year PFS and OS rates, respectively, in patients with T2–4N0–2M0 disease. Building on these findings, more intensive strategies have been investigated, including radiotherapy combined with chemotherapy, and ICIs. The NCT02621151 study (25) employed a quadruple regimen consisting of pembrolizumab, transurethral resection, concurrent pembrolizumab and gemcitabine, and radiotherapy, resulting in a 12-week CR rate of 83% and a one-year bladder-intact disease-free survival (BIDFS) of 77%. These findings indicate that radiotherapy combined with immunotherapy improves local disease control in MIBC.

In mUC, evidence supporting the combination of ICIs and radiotherapy remains limited. Sundahl et al. (26) reported an ORR of 44.4% and a mOS of 12.1 months in nine patients treated with concurrent pembrolizumab and SBRT to metastatic lesions. These patients had received first- to fourth-line therapy, and no clear benefit was observed in a cohort receiving sequential radiotherapy and immunotherapy. Sano et al. (27) retrospectively analyzed 36 patients with unresectable la/mUC who received second-line immunotherapy and reported a significant improvement in OS in patients receiving radiotherapy compared with those who did not (27.7 vs. 9.2 months), with a median radiotherapy dose of 58 Gy delivered in 13–30 fractions. Although statistical significance was not achieved because of limited sample size and treatment heterogeneity, these findings suggest that selected patients may benefit from this combined approach. Most available data involve second-line or later treatment, and the role of radiotherapy in first-line ICI–based therapy for mUC remains unclear.

This study explored the clinical benefit of incorporating radiotherapy after first-line systemic treatment in patients with la/mUC. A retrospective analysis was performed on patients treated with first-line ICIs combined with chemotherapy, with or without subsequent local radiotherapy, over eight years. PSM was employed to reduce selection bias, and subgroup and Cox regression analyses were conducted to identify populations most likely to benefit. Given the predominance of bladder cancer within urothelial carcinoma and its distinct clinical characteristics, the analysis focused primarily on this subgroup. The findings indicate that, among patients receiving first-line systemic therapy, the addition of radiotherapy before immunotherapy maintenance is associated with higher ORRs and significant prolongation of PFS, OS, and DOR, corresponding to a 56% reduction in the risk of disease progression and a 51% reduction in mortality risk. The study further explored practical considerations of this combined approach, including treatment timing, irradiation site, dose selection, patient selection, and safety, providing clinically relevant insights for future therapeutic optimization.

The sequencing of radiotherapy and ICIs may affect therapeutic efficacy (28). Most published retrospective studies in mUC have used radiotherapy before immunotherapy in second-line or later settings, except for the concurrent strategy reported by Sano et al. (29). Preclinical evidence supports a sequence-dependent interaction between radiotherapy and ICIs. Radiation-induced DNA damage can increase PD-L1 expression *via* ATM/ATR/Chk1-dependent signaling pathways within the DNA damage response, potentially improving subsequent responsiveness to ICIs. Some evidence suggests that administering PD-1 or PD-L1 inhibitors before radiotherapy should be approached with caution, as radiotherapy after immune checkpoint blockade may reduce the activity of tumor-infiltrating T cells already engaged in an anti-tumor immune response (30). In clinical practice, early integration of radiotherapy in mUC remains challenging because of extensive disease burden, including large primary tumor masses, pelvic or abdominal invasion, multiple visceral or osseous metastases, and serous cavity effusions. These findings indicate that early integration of radiotherapy may be feasible primarily in selected patients with limited metastatic burden.

In our study, most patients received radiotherapy after achieving disease stabilization following first-line chemo-immunotherapy. This approach enabled systemic therapy to reduce tumor burden, which may facilitate organ preservation and increase tumor antigen availability, improving the immune microenvironment. The reduction in irradiated target volume provided further benefits, including improved local disease control and lower incidence of treatment-related toxicity. Reduced tumor burden may also lessen hypoxia-related radioresistance and reduce the recruitment of immunosuppressive cell populations within the tumor microenvironment. The treatment protocol, which resumed ICI maintenance within 2–4 weeks after radiotherapy and continued for up to two years, shows current clinical practice and supports the feasibility of this sequential treatment strategy.

Regarding irradiation sites and target volume selection, radiotherapy has often been discussed for its potential abscopal effects, which have been mainly reported in the setting of visceral metastases. Brooks et al. (31) suggested that irradiation of multiple sites may enhance systemic anti-tumor immunity by increasing antigen release and promoting T-cell priming when radiotherapy is combined with ICIs. For primary lesions, Fukushima et al. (32) retrospectively analyzed 98 patients with inoperable la/mUC and found that radiotherapy to the primary site before pembrolizumab, compared with pembrolizumab alone, was associated with higher ORRs (64.7% vs. 18.5%), improved one-year PFS (52% vs. 28%), and higher OS rates (77% vs. 50%). However, Nishimura et al. (33) analyzed 30 patients with locally advanced disease and did not observe a significant benefit from adding concurrent chemoradiotherapy to pembrolizumab, highlighting the heterogeneity of treatment response and the importance of patient selection.

In our radiotherapy cohort, ten patients received irradiation to metastatic sites, whereas twenty underwent treatment to pelvic or bladder primary lesions with or without a small pelvic field. The overall local control rate reached 73.4%, with an 86.7% first-year local control probability. mPFS and mOS were significantly improved in the radiotherapy group, corresponding to a 56% reduction in the risk of disease progression and an approximately twofold increase in mDOR compared with the control group. Local failure within irradiated fields occurred in 26.6% of patients. Among those receiving pelvic radiotherapy, seven patients did not undergo small pelvic field irradiation because of extensive tumor burden, and four of these patients experienced early local recurrence. The remaining thirteen patients in the pelvic radiotherapy subgroup achieved a local control rate of 77%, whereas 76.9% of local failures in the immunotherapy-only group occurred within the first year of treatment. While palliative irradiation was delivered to over 50% the cohort, 14 patients received moderate-to-high-dose palliative intent to selected lesions only. Furthermore, an 85.7% rate of symptom relief in the radiotherapy group underscores the palliative value of treating symptomatic primary or metastatic lesions.

Radiation dose and fractionation are key determinants of immunomodulatory effects (34, 35). In bladder cancer models, a regimen of 10 Gy in two fractions showed synergistic anti-tumor effects when combined with PD-1 or PD-L1 blockade (36). In mUC, hypofractionated regimens, such as 24 Gy in three fractions and 60 Gy in ten fractions, delivered to metastatic lesions have been associated with a survival benefit when combined with immunotherapy (26, 37). Recent data presented at ASCO 2025 further support hypofractionated approaches in oligometastatic urothelial carcinoma (38), with Patrick Carriere et al. reporting that the addition of primary or metastatic radiotherapy (30 Gy in three fractions or 50 Gy in four fractions) resulted in a mPFS of 21 months and a mOS of 39 months. Swisher-McClure et al. (39) proposed that conventional fractionation of 1.8–2 Gy per fraction may be more appropriate for larger target volumes exceeding 5–10 cm³ to limit normal tissue toxicity. Several studies in mUC (32, 40, 41) have recommended conventionally fractionated radiotherapy (40–64 Gy in 20–32 fractions) combined with ICIs. The NRG Oncology/RTOG 0926 study (42) delivered 61.2 Gy in 34 fractions with concurrent chemotherapy for recurrent T1 bladder cancer, achieving bladder preservation in nearly 88% of patients at 3 years and a 5-year survival rate of 56.4%.

In this study, most patients received conventionally fractionated radiotherapy because of advanced disease burden. Only a limited number underwent hypofractionated treatment, precluding a comparative efficacy analysis. Four of six patients with bulky pelvic masses experienced in-field recurrence after conventional-dose radiotherapy ranging from 50 to 70 Gy. Three of these patients received total doses below 60 Gy, suggesting that higher biologically effective doses, potentially achieved through hypofractionation or advanced approaches such as spatially fractionated radiotherapy (SFRT), may warrant further investigation to improve outcomes in patients with large-volume disease.

Subgroup analyses indicated a higher OS benefit from the addition of radiotherapy in patients older than 65 years, those with postoperative recurrence, and those without liver metastases. Older patients may have limited tolerance to subsequent intensive systemic treatments, including antibody–drug conjugates combined with immunotherapy, chemotherapy, or intravesical therapy. In this context, well-tolerated local radiotherapy may serve as an effective consolidative option. Compared with synchronous metastatic disease, postoperative recurrence is generally associated with reduced sensitivity to first-line systemic therapy and a higher likelihood of primary or acquired resistance, partly due to tumor heterogeneity. The aggressive biological characteristics observed in this subgroup may be partially countered by radiotherapy through improvement of the local immune microenvironment and increased tumor immunogenicity, restoring treatment sensitivity and improving prognosis. Furthermore, the liver is recognized as an immune-privileged organ. In our cohort, liver metastasis was identified as an independent adverse prognostic factor for OS, with a 2.6-fold increase in mortality risk. Although radiotherapy directed at liver metastases has been shown to reduce CD8^+^ T cell apoptosis induced by immunosuppressive hepatic myeloid cells *via* the Fas–FasL pathway, thereby reversing systemic immunosuppression (43), irradiation of nonhepatic sites in patients with liver metastases may provide limited benefit, as suggested by the current findings.

Lung metastasis was identified as an independent adverse factor for PFS, increasing the risk of progression by approximately 1.1-fold. Radiotherapy improved clinical results in patients both with and without lung metastases, supporting its broader applicability in this subgroup. Furthermore, consistent with previous studies, elevated CRP levels were identified as an independent adverse prognostic factor for PFS. A meta-analysis demonstrated that CRP may reflect a pro-inflammatory tumor microenvironment in UC, and elevated CRP levels in patients receiving ICIs were associated with poorer OS, with a similar trend observed for PFS (44). However, subgroup analyses based on standard CRP cutoffs did not reveal statistically significant differences. Radiotherapy and achievement of CR or PR were identified as common, independent, favorable prognostic factors for both PFS and OS, suggesting that radiotherapy may improve long-term outcomes even in the absence of deep systemic responses. A trend toward improved outcomes was observed in patients who did not achieve CR or PR following first-line systemic therapy. In patients with suboptimal response to chemo-immunotherapy, radiotherapy may promote immunogenic modulation and facilitate conversion of immunologically “cold” tumors into more responsive phenotypes. No significant prognostic association was observed for lesion size, bone metastasis, or LDH levels. Biomarker analyses for PD-L1 and HER2 expression were limited by incomplete data availability.

Regarding safety, previous studies evaluating postoperative retroperitoneal lymph node metastases in urothelial carcinoma reported that grade 3 or higher adverse events were predominantly related to chemotherapy-induced hematologic toxicity, fatigue, and antibody–drug conjugate–associated sensory neuropathy, hyperglycemia, and diarrhea (45). Most radiotherapy-related adverse events were grade 1–2 gastrointestinal reactions. In the study by Sundahl et al., only one case of grade 3 lymphopenia was reported in patients receiving SBRT concurrently with immunotherapy. Across published urothelial carcinoma studies, grade 3 or higher adverse events were primarily hematologic or immune-related, with severe radiotherapy-associated toxicity being uncommon (19, 25, 26, 45). In our study, no treatment-related deaths occurred. Most adverse events were grade 1–2 and included hematologic toxicity, elevated transaminases, thyroid dysfunction, and infrequent immune-related events such as myocarditis, myelitis, and dermatitis, with no significant differences between treatment groups in grade 3–4 toxicity. Radiotherapy-related toxicities included radiation cystitis in 13.8% of patients, predominantly grade 1–2 with one grade 3 case, and grade 2 radiation enteritis in four cases; all were effectively managed with corticosteroids, antimicrobial therapy, and supportive care. In summary, integration of radiotherapy with immunotherapy represents a promising treatment strategy for advanced urothelial carcinoma. The study suggests that radiotherapy administered after first-line chemo-immunotherapy can prolong PFS and OS while maintaining an acceptable safety profile.

In summary, this propensity score−matched study evaluated the addition of local radiotherapy to first-line chemo-immunotherapy in patients with la/mUC. Although the findings may inform clinical decision-making, several methodological limitations should be acknowledged. First, this was a retrospective single-center study conducted over an eight-year enrollment period. Patient recruitment was challenging, resulting in a relatively small sample size. Although PSM was applied to reduce selection bias, residual confounding cannot be completely excluded, and prospective validation is required. Ongoing prospective trials, including NCT03486197 and NCT03150836, are expected to provide higher-level evidence. Second, the absence of statistically significant differences in ORR and DOR raises uncertainty regarding the effect of radiotherapy on early response endpoints and whether optimal benefit depends on irradiation site or fractionation strategy. Patients were treated by multiple clinical teams within a single center over an extended study period, resulting in heterogeneity in radiotherapy indications, target volumes, fractionation schedules, and prescribed doses. Furthermore, the relatively small sample size precluded stratified analyses of different radiotherapy modalities and limited detailed assessments of efficacy and safety across fractionation regimens. Moreover, analyses of PD-L1 and HER2 expression were limited by incomplete biomarker data, preventing evaluation of the predictive value of these markers for the combined radiotherapy and immunotherapy approach. Recent advances from studies such as RC48–016 and EV-302/KEYNOTE-A39 require a reassessment of the role of radiotherapy within evolving first-line treatment paradigms. Continued investigation is warranted to fully identify the characteristic of the patient most likely to benefit from radiotherapy and to define the therapeutic contribution of radiotherapy in mUC.

## 5 Conclusions

Extensive preclinical and clinical evidence supports further investigation of radiotherapy combined with ICIs as a therapeutic approach for advanced urothelial carcinoma. Multiple combination strategies, including antibody–drug conjugates and varying radiotherapy doses and fractionation schedules, are currently under active evaluation. Our study contributes to this field by showing that, in patients with recurrent or mUC, radiotherapy directed at primary or metastatic lesions, sequenced after first-line immunotherapy combined with chemotherapy, may extend PFS and OS with acceptable toxicity. Prospective clinical trials are required to provide more robust evidence and to elucidate the optimal integration, sequencing, and patient selection for this combined treatment strategy in advanced urothelial carcinoma.

## Data Availability

The original contributions presented in the study are included in the article/supplementary material. Further inquiries can be directed to the corresponding authors.
